# Clinical short-term outcomes of articular-sided and bursal-sided partial-thickness rotator cuff tears of less than 50% in a single surgeon series: A protocol of randomized controlled trial

**DOI:** 10.1097/MD.0000000000024965

**Published:** 2021-03-12

**Authors:** Jie Gu, Bo Dai, Xuchao Shi, Zhennian He

**Affiliations:** Department of Orthopedics, The People's Hospital of Beilun district of Ningbo, Zhejiang, China.

**Keywords:** arthroscopic repair, articular-sided, bursal-sided, clinical study protocol, Partial-thickness rotator cuff tears, random

## Abstract

**Background:**

There have been no published randomized clinical trial to assess the clinical outcomes between the articular-sided and bursal-sided tears. Therefore, a comparative analysis of evaluating and comparing the functional outcomes following arthroscopic repair of bursal-sided versus articular-sided partial-thickness rotator cuff tearsis essential.

**Methods:**

This study is a present randomized controlled trial which is conducted in our hospital. Consecutive patients with symptomatic articular-sided or bursal-sided partial-thickness rotator cuff tears underwent arthroscopic repair between June 2020 and January 2022. The institutional review board approved the study proposal (with number 10012030), and informed consent was obtained from all patients. Inclusion criteria were existence of an articular- or bursal-sided tear involving <50% of the tendon thickness—confirmed intraoperatively and treated with arthroscopic debridement with or without other decompression surgery (acromioplasty/distal clavicle resection)—and a minimum follow-up of 2 years. All patients followed the same postoperative rehabilitation program. The patients were assessed at baseline preoperatively, and at 1 year and 2 years postoperatively. Outcome parameters were measured at each respective follow-up, which included active range of motion in forward flexion and abduction of the affected shoulder, pain score as measured on the Numeric Pain Rating Scale, as well as outcome scores in terms of the Constant-Murley Score, and Oxford Shoulder Score.

**Results:**

Table 1 and Table 2 describe the data indicators that this article wants to evaluate and collect.

**Conclusions:**

We hypothesize that both groups of patients will show improvement in range of motion, functional outcome scores, and pain at 2 years, and that results would be similar between the two groups.

**Trial registration:**

This study protocol was registered in Research Registry (researchregistry6496).

## 1 Introduction

Partial-thickness rotator cuff tears (PTRCTs) can occur in different clinical situations and can be asymptomatic or cause pain and loss of function, affecting daily life. PTRCTs have a higher incidence rate compared with full-thickness tears, and interestingly, they tend to be more painful than full-thickness tears.^[1,2]^ Symptoms resulting from partial tears are thought to be the consequence of non physiologic tension created within the remaining intact rotator cuff fibers.^[3]^

Initial treatment of symptomatic PTRCTs is usually non-operative. Many PTRCTs can successfully be treated non-operatively with remission of symptoms after a period on physiotherapy accompanied by adequate pain medication. However, due to the hypo-vascularity in the critical zone of the tear site, and the effect of subacromial impingement, spontaneous healing with non-operative treatment has rarely been reported.^[4]^ Furthermore, the progression of symptomatic PTRCTs to fullthickness rotator cuff tears has been reported in up to 50% of cases. Surgical treatment is therefore indicated after failure of conservative management. Arthroscopic techniques have become the gold standard given the minimally invasive approach.^[5]^

PTRCTs could have a variety of possible etiologic factors, including tendon degeneration, acute trauma, accumulated microtrauma, and instability leading to secondary impingement. Articular-sided PTRCTs are thought to be a result of histologically demonstrable intrinsic tendon degeneration. Bursal-sided PTRCTs are thought to arise from both intrinsic and extrinsic factors, and are often contributed by subacromial impingement, with repetitive mechanical impingement of the rotator cuff tendon against the anterior third of the acromion.^[6]^ The difference in pathogenesis between articular-sided and bursal-sided tears could lead to a difference in healing potential, and therefore a possible difference in postoperative outcomes.^[7]^

To our knowledge, there have been no published randomized clinical trial to assess the clinical outcomes between the articular-sided and tears. Therefore, a comparative analysis of evaluating and comparing the functional outcomes following arthroscopic repair of bursal-sided versus articular-sided PTRCTs is essential. Based on the available literature, we hypothesize that both groups of patients will show improvement in range of motion, functional outcome scores, and pain at 2 years, and that results would be similar between the two groups.

## 2 Material and method

### 2.1 Study design

This study is a present randomized controlled trial which is conducted in our hospital. Consecutive patients with symptomatic articular-sided or bursal-sided PTRCTs underwent arthroscopic repair between June 2020 and January 2022. The institutional review board of the People's Hospital of Beilun district of Ningbo approved the study proposal (with number Z104930), and informed consent was obtained from all patients. The study protocol was registered in Research Registry (researchregistry6496).

### 2.2 Patients

Inclusion criteria were existence of an articular- or bursal-sided tear involving <50% of the tendon thickness—confirmed intraoperatively and treated with arthroscopic debridement with or without other decompression surgery (acromioplasty/distal clavicle resection)—and a minimum follow-up of 2 years. Tear depth was assessed with a probe and direct arthroscopic visualization.

Exclusion criteria included

(1) Ellman grade 1 PTRCTs (<3 mm deep), on the basis that this should be treated with debridement;
(2) Ellman grade 3 PTRCTs (>6 mm deep, >50% of the thickness), on the basis that this should be treated with repair;
(3) patients with both bursal- and articular-sided tears;
(4) patients with full-thickness rotator cuff tears requiring repair;
(5) suspected internal impingement (throwing athletes with anterior instability/anterior labral tear and PTRCTs);
(6) hyperlaxity (Beighton score >4) and/or patients thought to have anterior instability attributed to capsular laxity;
(7) other concomitant major surgery, such as superior labral anterior-posterior repair or biceps tenotomy/tenodesis; and
(8) previous surgery on the operative shoulder.

### 2.3 Randomization and allocation concealment

**Figure 1:**
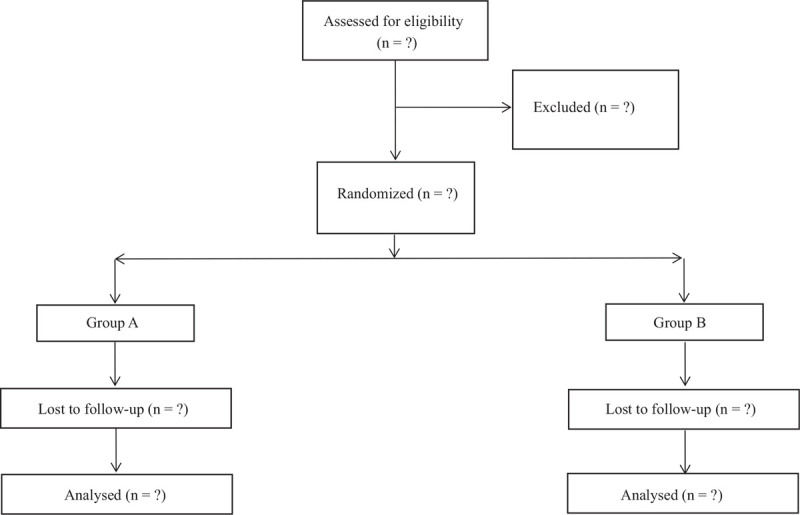
Consolidated Standards of Reporting Trials (CONSORT) diagram of patient flow through the study.

An independent statistician will obtain a computer-generated allocation sequence, which is kept in sequentially numbered opaque envelopes securely locked in a cupboard in the anesthetic room. The statistician who prepare the allocation sequence and the envelopes will be not involved in the recruitment process or the conduct of the study. All the patients will receive the same protocol for general anesthesia. Members who are not involved in the recruitment of the patients or assessing their pain postoperatively will carry out the sequential retrieval of the envelopes. The allocation sequence will be recorded by the anesthetist in a separate secure database. This is only revealed after the termination of the study and the analysis of the results (Fig. 1).

### 2.4 Surgical technique

All operations were performed with the patient in the beach-chair position under general anesthesia. The fibers of the anterior deltoid muscle were split after a skin incision approximately 5 cm long. Any acromioplasty was limited to the removal of osteophytes with a flat chisel. The coracoacromial ligament was released partially in all patients. All PTRCTs were repaired after conversion to a full-thickness tear by releasing the remaining tissue from the footprint. It is supposed to be difficult to determine the size and extension of the articular side tear at open surgery. The tear site could be detected by palpation because it felt softer than the surrounding tendon despite smooth appearance from the bursal side. Exact extension of the tear was determined after exploring the articular side. The tendon was repaired using the standard McLaughlin procedure. The bone trough was made at the foot-print, and the cuff was repaired to the greater tuberosity after the degenerative edge of the torn cuff was resected.

### 2.5 Rehabilitation program

All patients followed the same postoperative rehabilitation program. Patients were instructed to wear a shoulder brace with 0° of external rotation and 15° of abduction for 4 weeks. To prevent shoulder stiffness, pendulum exercise and passive gentle range of motion exercise were started whenever patients were tolerable to the postoperative pain. Active assisted range of motion exercise was allowed from 6 weeks, followed by resisted shoulder motion exercise from 3 months after operation.

### 2.6 Outcomes parameters

The patients were assessed at baseline preoperatively, and 1 year and 2 years postoperatively. The clinical assessments were conducted by a single and experienced physiotherapist from our department specifically tasked to assess and collect postoperative outcome measures. This assessor was blinded to the location of the partial-thickness rotator cuff tear repaired, and was an independent observer not involved in the clinical care of the patients. Outcome parameters were measured at each respective follow-up, which included active range of motion in forwarding flexion and abduction of the affected shoulder, pain score as measured on the Numeric Pain Rating Scale, as well as outcome scores in terms of the Constant-Murley Score, and Oxford Shoulder Score (Tables 1 and 2). Shoulder range of motion was measured using a Dual Digital Inclinometer (Dualer IQ Digital Dual Inclinometer-JTech Medical).

**Table 1:** Patient baseline demographics.

Demographics	Bursal-sided	Articular-sided	*P* value
Number of patients (knees)			
Age at surgery^∗^ (years)			
Male sex (no. [%])			
BMI^∗^ (kg/m^2^)			
Right side (no. [%])			
Follow-up^∗^ (years)			

BMI = body mass index.

∗ The values are given as the mean and the SD.

**Table 2:** Postoperative outcomes.

Outcomes	Bursal-sided	Articular-sided	*P* value
ROM			
CM score			
OSS			
NRS			

CM = Constant-Murley Score, NRS = Numeric Rating Scale, OSS = Oxford Shoulder Score, ROM = range of motion.

### 2.7 Statistical analysis

All data are expressed as mean ± SD. On several occasions the variances between groups are not homogenous, thus, we prefer the use of the Mann-Whitney *U* test to check the differences of numeric variables between groups. The Wilcoxon signed rank test is utilized to evaluate statistically significant differences pre and post-treatment. Categorical differences are tested via the Fischer exact method. All P-values are two-tailed and a value less than 0.05 are considered statistically significant. Statistical analysis is performed using SPSS 20.0 (SPSS Inc., Chicago, IL, USA).

## 3 Results

Tables 1 and 2 describe the data indicators that this article wants to evaluate and collect.

## 4 Discussion

To the best of our knowledge, there has been no published randomized clinical trial to assess the clinical outcomes between the articular-sided and tears. Therefore, a comparative analysis of evaluating and comparing the functional outcomes following arthroscopic repair of bursal-sided versus articular-sided PTRCTs is essential. Based on the available literature, we hypothesize that both groups of patients will show improvement in range of motion, functional outcome scores, and pain at 2 years, and that results would be similar between the two groups. There are several limitations to this study. We do not use a double-blind design or a placebo (ie, sham) group, as it is considered unethical by our institutional review board. Nonetheless, a blinded investigator is used to collect the postoperative data. Also, study participants are blinded as both interventions are done under general anesthesia. Of note, our results are specific to patients receiving general anesthesia, as the use of spinal anesthesia may provide different outcomes in the immediate postoperative period.

## Author contributions

**Conceptualization:** Jie Gu, Xuchao Shi.

**Data curation:** Jie Gu.

**Formal analysis:** Jie Gu, Bo Dai.

**Funding acquisition:** Zhennian He.

**Investigation:** Jie Gu, Bo Dai, Xuchao Shi.

**Methodology:** Bo Dai.

**Project administration:** Zhennian He.

**Software:** Bo Dai.

**Supervision:** Zhennian He.

**Validation:** Bo Dai.

**Visualization:** Xuchao Shi.

**Writing – original draft:** Jie Gu.

**Writing – review & editing:** Xuchao Shi, Zhennian He.
